# Cellular mRNA Activates Transcription Elongation by Displacing 7SK RNA

**DOI:** 10.1371/journal.pone.0001010

**Published:** 2007-10-10

**Authors:** Tara M. Young, Michael Tsai, Bin Tian, Michael B. Mathews, Tsafi Pe'ery

**Affiliations:** 1 Department of Biochemistry and Molecular Biology, New Jersey Medical School, Newark, New Jersey, United States of America; 2 Department of Medicine, New Jersey Medical School, Newark, New Jersey, United States of America; 3 Graduate School of Biomedical Sciences, University of Medicine and Dentistry of New Jersey, Newark, New Jersey, United States of America; National Cancer Institute, United States of America

## Abstract

The positive transcription elongation factor P-TEFb is a pivotal regulator of gene expression in higher cells. Originally identified in *Drosophila*, attention was drawn to human P-TEFb by the discovery of its role as an essential cofactor for HIV-1 transcription. It is recruited to HIV transcription complexes by the viral transactivator Tat, and to cellular transcription complexes by a plethora of transcription factors. P-TEFb activity is negatively regulated by sequestration in a complex with the HEXIM proteins and 7SK RNA. The mechanism of P-TEFb release from the inhibitory complex is not known. We report that P-TEFb-dependent transcription from the HIV promoter can be stimulated by the mRNA encoding HIC, the human I-mfa domain-containing protein. The 3′-untranslated region of HIC mRNA is necessary and sufficient for this action. It forms complexes with P-TEFb and displaces 7SK RNA from the inhibitory complex in cells and cell extracts. A 314-nucleotide sequence near the 3′ end of HIC mRNA has full activity and contains a predicted structure resembling the 3′-terminal hairpin of 7SK that is critical for P-TEFb binding. This represents the first example of a cellular mRNA that can regulate transcription via P-TEFb. Our findings offer a rationale for 7SK being an RNA transcriptional regulator and suggest a practical means for enhancing gene expression.

## Introduction

The positive transcription elongation factor b (P-TEFb) is a general transcription elongation factor and a protein kinase that phosphorylates the carboxy-terminal domain (CTD) of RNA polymerase II as well as other transcription factors [1], [2]. P-TEFb is composed of CDK9 and either cyclin T1, T2, or K. Cyclin T1 and CDK9 cooperate with the Tat protein and TAR RNA element of human immunodeficiency virus type 1 (HIV-1) to ensure processive transcription of the viral genome [3]–[5]. Numerous cellular proteins interact with P-TEFb [6]–[19] which appears to lie at the crossroads of multiple pathways including cell growth, differentiation, development, stress, apoptosis, and infection [13], [20]–[31]. P-TEFb is present in cells in an active form associated with the recruiting factor Brd4 [32], [33], and in repressed form associated with the hexamethylene bisacetamide (HMBA)-induced proteins HEXIM1 or HEXIM2 and 7SK RNA [9], [10], [27], [28], [34], [35]. HEXIM1 is an RNA binding protein [36] and 7SK is an abundant and evolutionary conserved small nuclear RNA (snRNA). The inhibitory complex, which engages ∼50% of P-TEFb and ∼30% of the cellular 7SK, is disassembled under stress conditions releasing P-TEFb to function in transcription. The 7SK/HEXIM/P-TEFb regulatory complex is strikingly reminiscent of the TAR/Tat/P-TEFb complex [37] although it blocks P-TEFb function. Mechanistically, the binding of 7SK and HEXIM decreases the kinase activity of P-TEFb and prevents its recruitment to the HIV-1 promoter. Hence, the 7SK/HEXIM/P-TEFb interaction may serve as a principal control point for the induction of cellular and HIV-1 viral gene expression during stress-related responses [10], [20], [27], [28], [38].

It is not known how P-TEFb is released from the inhibitory RNA-protein complex, but recent reports have identified new 7SK-containing complexes with RNA helicase A (RHA) and a number of hnRNP proteins [39], [40]. The level of 7SK in these complexes increases concomitantly with the disassembly of the 7SK/HEXIM/P-TEFb complex.

Here we report that RNA from the 3′-untranslated region (3'UTR) of the human I-mfa domain containing protein, HIC, is present in and regulates P-TEFb complexes. The C-terminus of HIC contains a cysteine-rich I-mfa domain which is 82 aa long and 74% identical to the corresponding region of the cellular protein I-mfa (inhibitor of MyoD family a) [41], [42]. We previously found that the HIC and I-mfa proteins interact with both the cyclin T1 subunit of P-TEFb and with HIV-1 Tat [12], [42]. These interactions are mediated by the proteins' homologous I-mfa domains, which are inhibitory to Tat and P-TEFb transcription. Paradoxically, however, HIC cDNA activates transcription from the HIV promoter in a fashion that is dependent on P-TEFb [12]. We analyze this discrepancy and demonstrate that the activation is mediated by HIC mRNA rather than HIC protein. The 3'UTR of HIC mRNA binds to and activates P-TEFb by displacing 7SK RNA from its complex with the elongation factor. This is the first example of a cellular mRNA that regulates elongation via interaction with P-TEFb complexes. Our findings provide a basis for understanding why this transcription factor is controlled by RNA molecules including 7SK and TAR. We anticipate that additional cellular mRNAs will be found to have a role in transcriptional regulation via P-TEFb.

## Results

### The 3'UTR of HIC is required to activate gene expression

**Figure 1:**
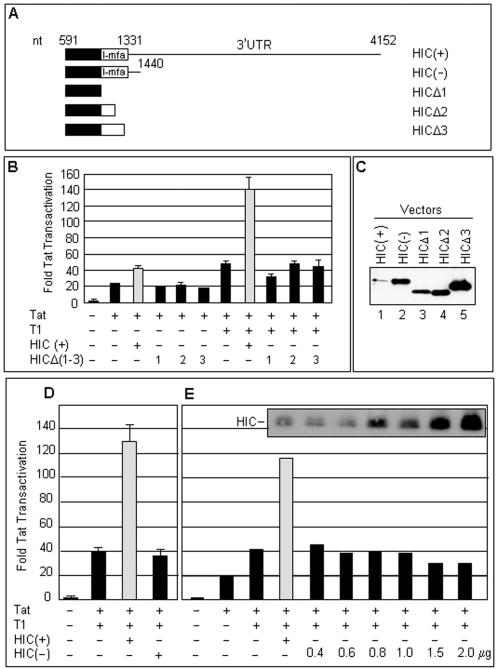
Activation of HIV-1 promoter by HIC cDNA requires its 3'UTR. (A) Schematic representation of HIC RNAs. The boxed area represents the protein coding region, including the C-terminal I-mfa domain. Full-length HIC cDNA is referred to as HIC(+). HIC(−) lacks all but the first 109 nt of the 2,821 nt HIC 3'UTR. (B) NIH 3T3 cells were co-transfected with the pcDNA3.1-FLAG-HIC vectors specified (2 µg), the HIV-1 LTR-firefly luciferase (100 ng) and CMV-Renilla luciferase (20 ng) reporter plasmids, pcDNA3.1-HA-Tat (5 ng) and pcDNA3.1-HA-cyclin T1 (T1; 100 ng) as indicated, and pcDNA3.1 empty vector to maintain a constant amount of DNA in each sample. Transactivation was measured at 24 hours post-transfection and expressed as fold Tat transactivation, calculated as the relative firefly:Renilla luciferase activity normalized to the value obtained without Tat, cyclin T1 or HIC. Data represent the average of three experiments with standard errors. (C) Expression of HIC protein. Extracts of cells (10 µg protein) transfected with Tat, cyclin T1 and the indicated HIC expression vectors were analyzed by western blotting using anti-FLAG antibody. (D) Tat transactivation was measured in the presence of HIC cDNAs carrying or lacking the 3'UTR. Assays were conducted as in panel B. (E) Reduced amounts of HIC(−) vector were compared to 2 µg of HIC(+) and assayed as in panel B. Data represent the average of two experiments. Inset: HIC protein in cell extracts monitored as in panel C.

The HIC protein interacts with viral and cellular transcription factors and regulates transcription driven by viral promoters [12], [42], [43]. HIC, and the I-mfa protein itself, modulate signal transduction pathways involved in cell fate, differentiation, and apoptotic events [44]–[48]. HIC interacts with P-TEFb by binding to its cyclin T1 subunit, and it also binds to HIV-1 Tat [12]. Similar results have recently been obtained with the I-mfa protein [42]. These interactions are all dependent on the I-mfa domain at the proteins' C-termini (Fig. 1A).

HIC cDNA (here called HIC(+)), activates Tat-mediated gene expression from the HIV-1 promoter in its long terminal repeat (LTR) [12]. Truncations within the HIC I-mfa domain (Fig. 1A) interacted and co-localized differentially with cyclin T1 and Tat. Removal of the I-mfa domain in whole (HICΔ1) or in part (HICΔ2 and Δ3) abrogated the interactions with cyclin T1, but the smaller I-mfa deletions retained some (HICΔ2) or all (HICΔ3) of their ability to co-immunoprecipitate and co-localize with Tat [12]. To determine whether the effect of HIC(+) on HIV LTR-driven gene expression correlates with the interaction of HIC with cyclin T1 and/or HIV-1 Tat, we tested these truncations in transient expression assays in 3T3 cells (Fig. 1B). In these cells, efficient Tat transactivation is dependent on the supply of human cyclin T1 which binds HIV-1 Tat and TAR better than its murine ortholog [49]. As seen previously [12], HIC(+) gave a 3–3.5 fold stimulation of Tat-mediated reporter gene expression in the presence of cyclin T1 and a ∼2 fold enhancement in its absence (Fig. 1B, gray bars). However, none of the HIC truncations stimulated HIV LTR-driven gene expression either in the presence or absence of cyclin T1 (Fig. 1B). These results appeared to be consistent with a requirement for direct interaction between the I-mfa domain of HIC and the cyclin T1 subunit of P-TEFb.

Immunoblot analysis showed that the C-terminally truncated proteins, which were expressed from constructs lacking the 3'UTR, accumulated to substantially higher levels than full-length HIC protein expressed from HIC(+) (Fig. 1C; compare lanes 3–5 with lane 1). Removal of most of the 3'UTR from the HIC cDNA, giving the HIC(−) construct (Fig. 1A), also led to elevated HIC protein levels (Fig. 1C, lane 2). Thus HIC protein expression is down-regulated by its 3'UTR, consistent with the presence of a regulatory element in the 3'UTR. This conclusion is supported by comparison of the levels of HICΔ1 protein generated from plasmids lacking and containing the 3'UTR (Figs. 1C and 3B).

**Figure 2:**
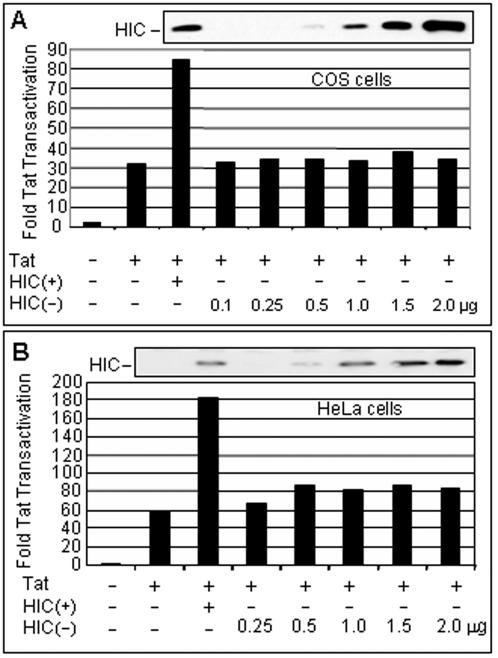
The 3'UTR of HIC is Necessary to Activate Tat Transactivation in HeLa and COS cells. (A) COS cells were co-transfected with the pcDNA3.1-FLAG-HIC vectors specified, the HIV-1 LTR-firefly luciferase (100 ng) and RSV-Renilla luciferase (100 ng) reporter plasmids, pcDNA3.1-HA-Tat (5 ng) and pcDNA3.1 empty vector to maintain a constant amount of DNA in each sample. Reduced amounts of HIC(−) vector were compared to 2 µg of HIC(+). Transactivation was measured at 24 hours post-transfection and expressed as fold Tat transactivation, calculated as the relative firefly:Renilla luciferase activity normalized to the value obtained without Tat and HIC. Data represent the average of two experiments. *Inset:* HIC protein in cell extracts monitored by western blotting using anti-FLAG antibody. (B) HeLa cells were co-transfected with pcDNA3.1-FLAG-HIC vectors specified, HIV-1 LTR-firefly luciferase (100 ng), CMV-Renilla luciferase (20 ng) reporter plasmids, pcDNA3.1-HA-Tat (5 ng) and pcDNA3.1 empty vector to maintain a constant amount of DNA in each sample. Reduced amounts of HIC(−) vector were compared to 2 µg of HIC(+). Transactivation was measured at 24 hours post-transfection and expressed as fold Tat transactivation, calculated as the relative firefly:Renilla luciferase activity normalized to the value obtained without Tat and HIC. Data represent the average of two experiments. *Inset:* HIC protein in cell extracts monitored by western blotting using anti-FLAG antibody.

Because the HIC(−) construct expresses the full-length HIC protein, we expected that like HIC(+) it would activate transcription from the HIV-1 promoter. Surprisingly, HIC(−) failed to activate expression of firefly luciferase from the HIV-1 promoter (Fig. 1D). In view of the high level of HIC protein expression from HIC(−), we entertained the possibility that this failure might be attributable to an excessive production of HIC protein by this plasmid. Transfection assays were conducted with a range of HIC(−) plasmid concentrations, encompassing amounts that gave rise to levels of HIC protein equal to that derived from HIC(+). In no case did HIC(−) stimulate expression from the HIV LTR (Fig. 1E). Similar results were obtained in the simian and human cells lines COS and HeLa (Fig. 2). These observations suggest that the 3'UTR of HIC cDNA is necessary for HIC to activate HIV gene expression.

Sequences in mRNA 3'UTRs that determine the site of translation of an mRNA can influence the function of the resultant protein [50], but several experiments argue that such phenomena do not account for the findings illustrated in Figure 1. First, the intracellular distribution of HIC generated from the HIC(−) plasmid was similar to that of HIC from HIC(+), and it co-localized with cyclin T1 in nuclear speckles and with HIV-1 Tat in the nucleolus to the same extent as HIC from the HIC(+) plasmid (data not shown, [12]). Second, HIC protein interacted with cyclin T1 (and indirectly with CDK9) and HIV-1 Tat in co-immunoprecipitation experiments regardless of whether it was expressed from cDNA containing or lacking the 3'UTR [12], [42]. Third, HIC produced in *E. coli* as a GST fusion protein interacted with both cyclin T1 and P-TEFb from HeLa cell extracts [12]. We conclude that the inability of HIC(−) to activate gene expression is probably not due to a failure of HIC protein to localize correctly or to interact with P-TEFb or Tat when expressed from a cDNA lacking the 3'UTR.

### The HIC 3'UTR is sufficient to activate gene expression

**Figure 3:**
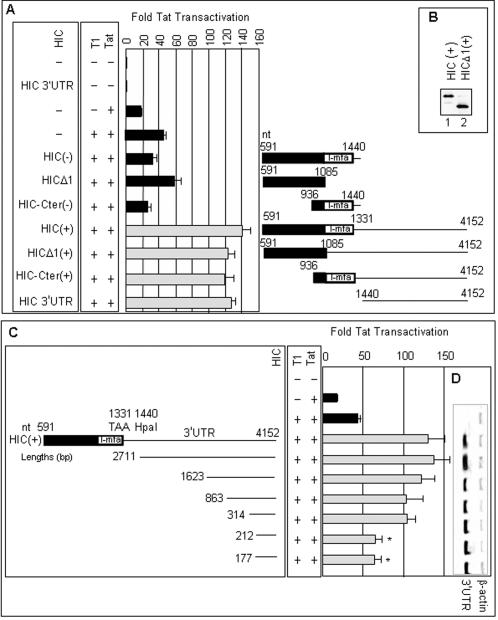
The HIC 3'UTR fragments are sufficient to increase gene expression. (A) NIH 3T3 cells were transfected with the pcDNA3.1-FLAG vectors indicated, together with other plasmids as in Fig. 1B. The pcDNA3.1-FLAG vectors expressed regions of the HIC protein and cDNA shown. Note that the HICΔ1(+) construct is identical to HIC(+) except for a point mutation that truncates the protein immediately before its I-mfa domain. Data represent the average of three experiments with standard errors and are presented as in Fig. 1B. (B) HIC protein expression was monitored as in Fig. 1C. (C) Deletions expressing the indicated regions of the 3'UTR were assayed as in panel A. Asterisks (*) indicate that the activities of the two shortest fragments were statistically different from the full-length 3'UTR (P = 0.0188 and 0.0181, respectively, in a Student's t-Test (two-tailed distribution, two-sample unequal variance)). (D) Total RNA was analyzed by relative quantitative RT-PCR using primers against the 3′ end of the HIC 3'UTR, and β-actin mRNA as an internal standard, producing fragments of 101 and 294 bp, respectively.

To evaluate the contribution of the HIC protein coding sequence to the HIC 3'UTR-dependent activation of gene expression, we tested two further pairs of plasmids expressing different parts of the protein from vectors that either contain or lack the HIC 3'UTR (Fig. 3A). As a partner to HICΔ1 which lacks both the I-mfa domain and 3'UTR, we generated HICΔ1(+). This plasmid is identical to HIC(+) except that it has a point mutation which introduces a stop codon at amino acid 164, immediately before the I-mfa domain. We also deleted the N-terminal half of the HIC protein coding sequence, yielding HIC C-terminal constructs that possess or lack the 3'UTR (HIC-Cter(+) and HIC-Cter(−)).

These vectors stimulated expression from the HIV promoter only if they contained the HIC 3'UTR, irrespective of the nature of the proteins encoded (Fig. 3A). Remarkably, the I-mfa domain, which interacts with cyclin T1 and Tat, was not required as shown by comparing the activity of the vectors HIC-Cter(−) and HIC-Cter(+) or HICΔ1 and HICΔ1(+) (Fig. 3A). We next determined whether the protein coding region is entirely dispensable for this activity, by testing a vector that expresses the 3'UTR alone. As seen in Figure 3A, this construct activated Tat- and cyclin T1-mediated reporter gene expression by ∼3 fold, just as well as HIC(+). The 3'UTR did not activate expression in the absence of Tat and human cyclin T1 (Fig. 3A), thereby excluding general effects on expression from the HIV LTR. We deduce that the 3'UTR of HIC is sufficient as well as necessary for the Tat- and cyclin T1-mediated activation of gene expression from the HIV-1 LTR.

The HIC 3'UTR is long, ∼2.8 kb, compared to a mean of ∼1.0 kb for human mRNAs [51]. To address the minimal fragment size that retains activity, we generated a nested set of truncations containing the 3′ end of the 3'UTR (Fig. 3C). A 3'UTR fragment 1623 nucleotides (nt) in length gave a ∼3 fold activation, similar to that elicited by HIC(+) cDNA or its almost full-length 3'UTR (2,712 nt). Truncations producing fragments of 863 and 314 nt, gave an activation of ∼2.5 fold, which was not statistically different from that of the full-length 3'UTR. Fragments of 212 and 177 nt elicited a lesser stimulation, ∼1.75 fold (Fig. 3C). Analysis by semi-quantitative reverse transcription followed by polymerase chain reaction (RT-PCR) demonstrated that RNA was produced from each truncation at roughly the same levels (Fig. 3D). These data show that specific short fragments of the 3'UTR can activate gene expression.

### The 3'UTR of HIC activates transcription from the HIV LTR

**Figure 4:**
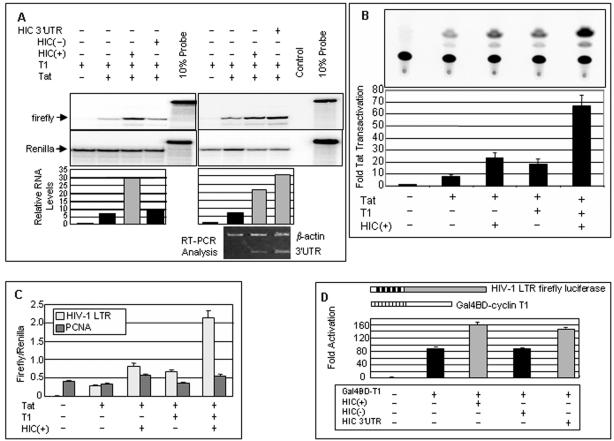
The HIC 3'UTR acts at the transcriptional level. (A) NIH 3T3 cells were co-transfected with the two firefly luciferase reporter plasmids, and vectors expressing Tat, cyclin T1 and the indicated HIC vectors as in Fig. 1B. RNA was isolated from nuclear fractions 24 hours post-transfection, subjected to RNase protection assays [12] and hybridized to probes for firefly luciferase RNA *(top),* or Renilla luciferase RNA to control for transfection efficiency *(bottom).* The control lacked cellular RNA but was digested with RNases A and T1. The probe lane contains 10% of the input probe RNA. Arrows indicate the position of protected luciferase probes. Bar graphs represent data averaged from two experiments calculated as relative firefly:Renilla RNA levels normalized to the sample with cyclin T1 but not Tat or HIC. Relative quantitative RT-PCR was performed as in Fig. 3D to examine HIC 3'UTR and β-actin mRNA levels. (B) HIC Activates Tat-mediated CAT expression. NIH 3T3 cells were co-transfected with the reporter plasmids HIV-1-CAT (100 ng) and CMV-Renilla luciferase (20 ng), and the plasmids pcDNA3.1-HA-Tat (5 ng), pcDNA3.1-HA-cyclin T1 (T1) (100 ng), 2 µg pcDNA3.1-HIC(+) expression vector, or pcDNA3.1 empty vector as indicated. Transactivation was measured at 24 hours post-transfection and expressed as fold Tat transactivation, calculated as the relative CAT:Renilla luciferase activity normalized to the value obtained without Tat, cyclin T1 and HIC. Data represent the average of three experiments with standard errors. (C) The PCNA promoter is only slightly responsive to HIC(+). NIH 3T3 cells were co-transfected with100 ng HIV-1 LTR-firefly luciferase (light bars) or PCNA-firefly luciferase (dark bars) and other vectors as in Fig. 1B. (D) Tat is dispensable in the tethering system. NIH 3T3 cells were co-transfected with Gal4BD-HIV-1 LTR-firefly luciferase reporter plasmid and Gal4BD-T1 expression vector (100 ng each), 20 ng CMV-Renilla luciferase reporter plasmid, 5 ng pcDNA3.1-HA-Tat expression vector, and 2 µg pcDNA3.1 vectors expressing FLAG-HIC(+), FLAG-HIC(−) or the HIC 3'UTR alone as indicated. Data represent the average of three experiments with standard errors, presented as fold activation by Gal4BD-T1 at 24 hours calculated as the relative firefly:Renilla luciferase activity normalized to the value obtained without Gal4BD-T1, HIC or Tat. The schematic diagrams the HIV-1 LTR-firefly luciferase construct furnished with five upstream Gal4 sites, and the Gal4BD-cyclin T1 fusion protein (Gal4BD-T1).

RNase protection assays were carried out to ascertain whether stimulation of gene expression by the HIC 3'UTR reflects an action at the RNA level. As expected, Tat (in the presence of human T1) increased the abundance of luciferase transcripts from the HIV LTR-firefly luciferase construct, whereas transcripts from the control vector (CMV-Renilla luciferase) were unaffected (Fig. 4A, left). A further increase in firefly, but not Renilla, luciferase transcripts was elicited by HIC(+) but HIC(−) had little or no effect. The HIC 3'UTR alone was sufficient to increase firefly luciferase RNA (Fig. 4A, right). RT-PCR analysis demonstrated that comparable amounts of 3'UTR RNA were produced from the HIC(+) and HIC 3'UTR plasmids (Fig. 4A, bottom right). In other experiments, the 314 nt 3′-terminal fragment of the 3'UTR also increased the level of firefly luciferase transcripts (data not shown). Thus, the HIC 3'UTR is both necessary and sufficient to increase firefly luciferase reporter RNA levels driven by the HIV-1 LTR. Similar results were obtained in HeLa and COS cells (data not shown).

Next we tested the effect of HIC(+) on a different reporter driven by the HIV-1 LTR. In 3T3 cells, expression of the chloramphenicol acetyltransferase (CAT) from the HIV LTR was also enhanced ∼3 fold by HIC(+) (Fig. 4B) while expression of firefly luciferase from the PCNA promoter, which is only weakly dependent on P-TEFb [11], was only mildly stimulated by HIC(+) (Fig. 4C) or the HIC 3'UTR alone (data not shown). These results argue against effects on protein or mRNA stability and strongly suggested that the 3'UTR functions at the level of transcription. Tat is an RNA-binding transcriptional activator that functions by recruiting P-TEFb to the HIV promoter [52]. To determine whether this viral protein is required for activation of gene expression by the HIC 3'UTR, we took advantage of a modified HIV promoter that can bind cyclin T1 in the absence of Tat. The HIV-1 LTR-firefly luciferase construct is furnished with Gal4 binding sites upstream of the viral promoter. When co-transfected with a vector encoding a Gal4 binding domain (Gal4BD)-cyclin T1 fusion protein, P-TEFb is artificially tethered to the HIV-1 LTR [53]. In this system, expression from the HIV-1 LTR was stimulated ∼2 fold by HIC(+) or by the isolated HIC 3'UTR but not by HIC(−) (Fig. 4D). These data implicated P-TEFb in the response of the HIV-1 promoter to the HIC 3'UTR.

### The 3'UTR of HIC binds P-TEFb and displaces 7SK RNA

**Figure 5:**
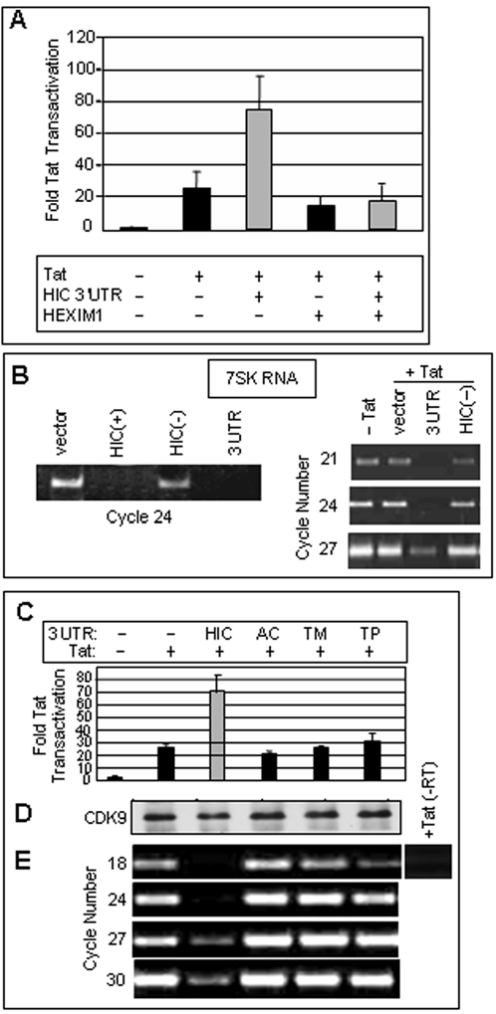
The HIC 3'UTR releases 7SK RNA from P-TEFb complexes. (A) HEXIM1 antagonizes activation by the HIC 3'UTR. HeLa cells were co-transfected in the presence or absence of pFLAG-CMV2-HEXIM1 (2 µg). Other plasmids and experimental details were as in Fig. 1B. Data represent the average of five experiments with standard errors. (B) HIC 3'UTR is sufficient to release 7SK from P-TEFb. HeLa cells were transfected with 100 ng HIV-1 LTR firefly luciferase and 20 ng CMV Renilla luciferase, with or without 5 ng HIV-1 Tat and 2 µg pcDNA3.1 empty vector, HIC(+), HIC(−) or the 3'UTR alone as indicated. Cells were harvested 24 hours post-transfection and assayed for firefly and Renilla luciferase activity to verify activation by HIC(+) and the 3'UTR (data not shown). P-TEFb was immunoprecipitated from cell extracts using antibody against CDK9. RNA was extracted from immunoprecipitates and subjected to RT-PCR using primers against 7SK RNA. The number of PCR cycles is specified for each experiment. (C) The 3'UTR of HIC specifically activates gene expression. HeLa cells were transfected as in panel A, and with 2 µg of vectors expressing the 3'UTR of cardiac actin (AC), tropomyosin (TM), or troponin (TP) as controls. Cells were harvested 24 hours post-transfection and assayed for firefly and Renilla luciferase activity. Data are represented as firefly/Renilla activity normalized to the sample without Tat. (D) Cell extracts were immunoblotted and probed with antibody to CDK9. (E) HIC 3'UTR specifically releases 7SK RNA from P-TEFb. Immunoprecipitated P-TEFb was assayed for 7SK RNA as in panel B. The number of PCR cycles is specified.

P-TEFb complexes of two types have been identified: inactive complexes contain HEXIM proteins and 7SK RNA, both of which are absent from active complexes. The release of 7SK RNA, resulting in the activation of P-TEFb, has been observed as a response to stress [20], [27], [28]. We therefore considered the possibility that the HIC 3'UTR may activate gene expression by displacing 7SK RNA. First, to determine whether HIC 3'UTR functions through the P-TEFb/7SK/HEXIM mechanism, we examined the effect of HEXIM1 on the response to the HIC 3'UTR (Fig. 5A). Overexpression of HEXIM1 caused a small inhibition of Tat-dependent gene expression, presumably by decreasing the availability of active P-TEFb [34], [54]–[56], but almost completely suppressed the ability of the 3'UTR to enhance Tat transactivation (Fig. 5A). The observation that the effect of the HIC 3'UTR is antagonized by HEXIM1 suggested that it targets the same complex. To test this hypothesis, we next investigated the effect of HIC 3'UTR on the binding of 7SK RNA to P-TEFb. P-TEFb was immunoprecipitated with anti-CDK9 antibody and analyzed for its 7SK RNA content by RT-PCR after transfection of HIC(+) or HIC 3'UTR which activate transcription, or HIC(−) that has no effect on transcription (Fig. 5B). Transfection of HIC(+) or its 3'UTR reduced the amount of 7SK RNA immunoprecipitated with P-TEFb, whereas HIC(−) did not (Fig. 5B, left). Semi-quantitative analysis indicated that the transfected HIC 3'UTR caused a 20–30 fold reduction of P-TEFb-associated 7SK (Fig. 5B, right). As additional controls, we tested the 3'UTRs of cardiac actin, tropomyosin and troponin (AC, TM, and TP), all of which failed to stimulate luciferase expression from the HIV-1 LTR in transactivation assays (Fig. 5C). Expression of the 3'UTRs was verified by RT-PCR (data not shown). Immunoprecipitated P-TEFb from these transfections contained approximately equal amounts of CDK9 (Fig. 5D), but only the HIC 3'UTR displaced 7SK RNA from the complex (Fig. 5E).

**Figure 6:**
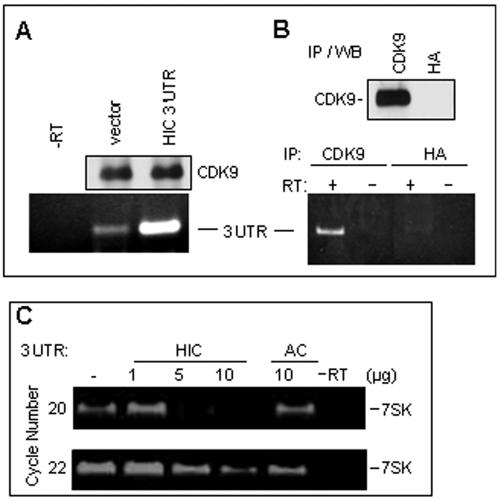
The HIC 3'UTR binds to P-TEFb. (A) Endogenous HIC 3'UTR binds to P-TEFb and overexpression of the 3'UTR results in an increased amount of HIC 3'UTR RNA bound to P-TEFb. HeLa cells were transfected with 2 µg of empty vector or HIC 3'UTR. RNA from immunoprecipitated P-TEFb was assayed by RT-PCR using primers for the 3′ end of the HIC 3'UTR as in Fig. 3D. (B) Endogenous HIC 3'UTR binds P-TEFb. P-TEFb was immunoprecipitated from HeLa cells using antibody against CDK9. Antibody against HA was used as a negative control (*upper panel*). Immunoprecipitates were examined for HIC RNA as in panel A. (C) Displacement of 7SK in vitro. HeLa cell nuclear extract [27] was incubated in the presence of the indicated amounts of in vitro transcribed HIC 3'UTR or cardiac actin (AC) 3'UTR. P-TEFb was immunoprecipitated using antibody against CDK9, RNA was extracted from the immunoprecipitates and subjected to RT-PCR using primers against 7SK RNA.

If the HIC 3'UTR replaces 7SK RNA in P-TEFb complexes, we would expect to find the HIC 3'UTR in such complexes. Analysis of anti-CDK9 immunoprecipitates by RT-PCR showed that HIC RNA was present in P-TEFb complexes from cells transfected with empty vector, and at much higher levels (>10,000-fold more) in complexes from cells overexpressing the 3'UTR (Fig. 6A). The 3'UTR was present in P-TEFb immunoprecipitates from untransfected cells but was not detected when immunoprecipitation was conducted with an irrelevant antibody (anti-HA; Fig. 6B). Estimation of P-TEFb–associated RNAs indicated that in transfected cells the 3'UTR was present at a level slightly higher than 7SK RNA in control cells. In further experiments the HIC coding region was also detected (data not shown). Thus, both endogenous and exogenous HIC mRNA is associated with P-TEFb in cells.

Finally, we sought direct evidence that the HIC 3'UTR can displace 7SK RNA from P-TEFb complexes. The HIC 3'UTR, synthesized in vitro using T7 RNA polymerase, was incubated with nuclear extract from HeLa cells and P-TEFb immunoprecipitates were examined for 7SK RNA. As in transfected cells, the HIC 3'UTR displaced 7SK RNA from the P-TEFb complexes whereas a control RNA (AC 3'UTR) synthesized in the same way failed to do so (Fig. 6C). Taken together, these findings support the conclusion that the HIC 3'UTR activates gene expression by displacing 7SK from repressed P-TEFb complexes.

## Discussion

Multiple functions have been found to reside in mRNA 3'UTRs, including elements governing RNA stability, localization and translation. Our work shows that the unusually long HIC 3'UTR harbors a novel function which stimulates transcription via P-TEFb by displacing 7SK. This finding reconciles the observations that transfected HIC cDNA stimulates P-TEFb–dependent gene expression whereas the HIC and I-mfa proteins are inhibitory in a cell-type dependent manner [12], [42]. More importantly, it suggests a mechanism for release of P-TEFb from inhibitory complexes. For example, in stress-induced cardiac hypertrophy or after exposure to UV radiation or transcription inhibitors, increased P-TEFb activity correlates with decreased 7SK RNA binding [20]. The mechanisms whereby these stress signals reach the 7SK-containing P-TEFb complex are not known. Our observations raise the possibility that RNA sequences in mRNAs or in other RNA molecules, function like the HIC 3'UTR to activate P-TEFb.

### A predicted structural element in HIC 3'UTR

**Figure 7:**
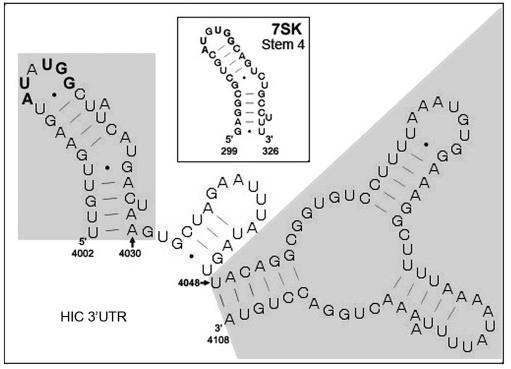
Similarity in RNA structure between the 3′ terminal region of HIC mRNA and regions in 7SK RNA. The 314 nt 3′ terminal region of the HIC 3′ UTR (nt 3,839–4,152) was aligned with the human 331 nt 7SK sequence using Foldalign. Two regions of HIC with significant score are shaded in gray. Region 1 was predicted to be structurally similar to stem 4 of 7SK (boxed) and region 2 to an alternative form of 7SK stem 2.

7SK serves as a structural scaffold for HEXIM1 and P-TEFb in the inhibitory complex, as well as for the binding of other proteins. Recent investigations have revealed a high degree of specificity in the interaction of cellular proteins with structural elements of 7SK. For example, the single-stranded RNA binding proteins hnRNPA1 and A2 bind exclusively to stem 3 of 7SK while hnRNP R and Q1 bind stems 1 and 3 [40]. Stem 1 is also important for the binding of RHA [40]. An extensive structure-function study led to the demonstration that both the 5′- and 3′-terminal stem-loop structures of 7SK are required for binding P-TEFb in vivo. The 5′ stem-loop structure (stem 1) is necessary for binding HEXIM1, which is a prerequisite for binding P-TEFb; in the next step, the 3′-terminal hairpin of 7SK (stem 4; [57]) interacts with P-TEFb [37]. We therefore used bioinformatic tools to examine the terminal segment of the HIC 3'UTR for the existence of an imperfect stem-loop structure that resembles stem 4 of 7SK [57]. A similar hairpin structure is predicted within the HIC 3'UTR (nt 4002–4030; Fig. 7, shaded area 1). The loop region of this hairpin contains a sequence, AUPuUGG, that is shared with stem 4 of 7SK RNA (Fig. 7, inset). A neighboring sequence in the HIC 3'UTR (nt 4048–4108 shaded area 2) is predicted to form a structure similar to that predicted for stem 2 of 7SK which contributes to, but is not essential for, its binding to P-TEFb [37], [57]. Both structures are present in the 314-nt fragment which retains full activity in stimulating HIV gene expression. They are also present in the shorter fragments of 212 and 177 nt, which display partial activity (Fig. 3C), suggesting that additional sequences contribute to the 3'UTR's activity. No similar structure was predicted for the AC 3'UTR which did not displace 7SK in vivo or in vitro. Such structures present in the 3'UTRs of other mRNAs may exert the same function. Future structure-function analysis of the 314-nt fragment will define the RNA elements that are necessary for HIC 3'UTR function.

### Multiple complexes balance the function of P-TEFb

The P-TEFb inhibitory complex has been studied in detail. It was established that the binding of HEXIM1 to 7SK RNA is required for its inactivating interaction with P-TEFb. Only about 20% of HEXIM1 is bound to 7SK [35], and it has recently been shown that most of HEXIM1 is complexed with unidentified cellular RNAs in vivo [36]. Similarly, 7SK was recently reported to reside in a variety of RNA-protein complexes (RNPs) containing for example hnRNPs [39], [40]. Based on our observation that P-TEFb interacts with elements in the HIC 3'UTR, we consider it likely that multiple P-TEFb–containing RNPs exist. Microarray analysis could be used to define the set of mRNAs and non-coding RNAs that are associated with P-TEFb. We speculate that the interaction of this transcription factor with mRNAs, or their precursors, is an evolutionarily ancient aspect of transcriptional control. Furthermore, we speculate that different sets of RNA ligands could be present under various physiological conditions and states of differentiation, conferring the ability to fine-tune the regulation of this essential transcription factor. P-TEFb binds RNA polymerase II and phosphorylates the CTD which is involved in many aspects of transcription and RNA metabolism [58]. P-TEFb itself is involved in transcription initiation, capping, splicing and 3′ end formation in mammalian, insect and yeast systems [19], [59]–[66] and increasing numbers of cellular proteins are found to interact with P-TEFb [67]. Thus, P-TEFb–containing RNPs could have a role in various aspects of RNA processing as well as in transcription itself.

### HIC protein and mRNA

We initially isolated HIC as a protein that binds the cyclin T1 component of P-TEFb. Subsequently, we found that over-expression of its I-mfa domain inhibits P-TEFb-dependent gene expression. In addition, HIC possesses RNA binding activity via its conserved basic region [42] (Q. Wang, et al. unpublished results). It is tempting to speculate that the HIC protein and the 3'UTR of its mRNA interact with P-TEFb in a fashion reminiscent of the Tat/TAR/P-TEFb and HEXIM/7SK/P-TEFb interactions. In these well-studied instances, RNA molecules are key participants in the positive and negative regulation of P-TEFb function, respectively. Interestingly Tat was recently demonstrated to bind 7SK and disrupt the inhibitory P-TEFb complex [56], [68]. It remains to be determined whether HIC can also bind to 7SK and disrupt its interaction with HEXIM, thereby releasing P-TEFb. While the physiological roles of HIC are not yet well understood, its expression, like that of its *Xenopus* ortholog XIC, appears to be under tight control [42], [48] (Q. Wang et al., unpublished results). The HIC and XIC proteins, which are active during differentiation and developmental scenarios, could activate P-TEFb via interactions with cyclin T1 and the HIC 3'UTR causing a reduction of the inhibitory complex [69], [70]. Conceivably this action could be restricted to the vicinity of the transcription apparatus at specific genes. Documentation of the existence of such complexes is an important next step in evaluating this model.

Finally, our findings suggest a general mechanism whereby gene expression can be controlled at the level of P-TEFb by signals transmitted through mRNA as well as protein modulators. Exploitation of this phenomenon to increase transcription and gene expression by transfection of the HIC 3'UTR, or its derivatives, could be useful when enhanced yields of RNA or protein is required either in commercial or laboratory settings.

## Materials and Methods

### Plasmids and Plasmid Construction

A plasmid containing the 3'UTR of HIC was generated by sub-cloning the HpaI/XhoI fragment of HIC(+) into pcDNA3.1. All HIC constructs and additional plasmids for expression in mammalian cells were generated or obtained as previously described [12].

### Cell Culture

NIH 3T3, COS7 and HeLa cells were obtained from American Type Culture Collection and maintained in Dulbecco's Modified Eagle's Medium (Invitrogen Corp., Carslbad, CA) supplemented with 10% heat inactivated fetal bovine serum.

### Gene Expression Assays

NIH 3T3 (1×10^5^ cells), COS (1.6×10^5^ cells) and HeLa cells (1.6×10^5^) were seeded in 6-well dishes and transfected 24 hr later using Lipofectamine 2000 (Invitrogen). Cells were harvested 24 hr post-transfection and lysed in 300 µl of passive lysis buffer according to the manufacturer's instructions (Promega Corp., Madison, WI) for luciferase assays. Lysates were clarified by centrifugation and assayed for luciferase activity using the Promega dual luciferase reporter system according to the manufacturer's instructions.

### Western Blotting

NIH 3T3 or HeLa cells were seeded, transfected and harvested for gene expression assays. Cell extracts (10 µg protein) were separated by denaturing polyacrylamide gel electrophoresis and subjected to western blotting as described [71]. Western blots were probed for FLAG-HIC using anti-FLAG antibody (Sigma-Aldrich, St. Louis, MO) or for CDK9 using anti-CDK9 antibody (Santa Cruz Biotechnology, Inc., Santa Cruz, CA).

### Relative Quantitative Reverse Transcription-Polymerase Chain Reaction (RT-PCR)

Total RNA was purified from NIH 3T3 cells, using Trizol (Invitrogen) according to the manufacturer's protocol. Total RNA (2 µg) was subjected to relative quantitative RT-PCR performed using Quantum RNA β-actin internal standards coupled with RT-PCR (Ambion). Primers used (sequence available from the authors upon request) amplify a 101 bp region in the 3′ end of the 3'UTR. PCR products were separated in a 1% agarose gel and stained with SYBR Green (Molecular Probes, Inc., Eugene, OR). P-TEFb-associated RNA levels were compared using an amplification efficiency value of 70%.

### Immunoprecipitation and RNA Isolation

HeLa cells were seeded at 1.6×10^5^ cells per well in a 6-well dish and transfected 24 hr later using Lipofectamine 2000 (Invitrogen). Cells were harvested 24 hr post-transfection in a high salt buffer containing 25 mM Tris pH 8.0, 250 mM NaCl, 1mM EDTA, 1% NP40, 0.5 mM PMSF, 0.5 mM DTT and 40 U RNasin. Extracts were homogenized through a narrow gauge syringe, incubated for 10 min on ice and clarified by centrifugation at 13,000×g for 10 min at 4°C. Immunoprecipitation was performed with protein A-Sepharose beads and 2 µl rabbit anti-CDK9 antibody (Santa Cruz Biotechnology Inc., Santa Cruz, CA) for 2 hr at 4°C. Immunoprecipitates were washed 3 times in high salt buffer, and beads were heated to 100°C in a buffer containing 25 mM Tris pH 7.6, 250 mM NaCl, 0.5% SDS and 5 mM EDTA for 5 min. Half of the mixture was heated with 6×Laemmli sample buffer, resolved in an SDS-polyacrylamide gel, transferred to nitrocellulose and probed with antibody to CDK9. The remaining beads were pelleted and RNA was isolated from the supernatant using Trizol (Invitrogen), according to the manufacturer's instructions. RT-PCR was performed using the Retroscript kit (Ambion) with primers directed toward the 3'UTR as above and toward 7SK RNA which amplify the entire 331 bp of 7SK RNA [20].

### RNA Structure Prediction

7SK (NR_001445) and HIC (NM_199072) 3'UTR sequences were acquired from NCBI. The 314 nt HIC sequence, nt 3,806–4,152, was divided into 4 overlapping segments of 150 nt each at 75 nt intervals. Each segment was aligned against 7SK using the RNA structure alignment tool Foldalign [72] with the maximum motif length set to 100 and default sequence length difference settings. Foldalign was also used to derive a structure for the 314 nt segment based on its alignments with 7SK RNA.
